# Supplementation of 1-Kestose Modulates the Gut Microbiota Composition to Ameliorate Glucose Metabolism in Obesity-Prone Hosts

**DOI:** 10.3390/nu13092983

**Published:** 2021-08-27

**Authors:** Ayako Watanabe, Takumi Tochio, Yoshihiro Kadota, Motoki Takahashi, Yasuyuki Kitaura, Hirohito Ishikawa, Takanori Yasutake, Masahiro Nakano, Hiroe Shinohara, Toru Kudo, Yuichiro Nishimoto, Yoshinori Mizuguchi, Akihito Endo, Yoshiharu Shimomura

**Affiliations:** 1 Laboratory of Nutritional Biochemistry, Department of Applied Biosciences, Graduate School of Bioagricultural Sciences, Nagoya University, Nagoya 464-8601, Aichi, Japan; motokit9@gmail.com (M.T.); ykitaura@agr.nagoya-u.ac.jp (Y.K.); 2 Department of Gastroenterology and Hepatology, Fujita Health University, Toyoake 470-1192, Aichi, Japan; t-tochio@bfsci.co.jp; 3 B Food Science Co., Ltd., Chita 478-0046, Aichi, Japan; y-kadota@bfsci.co.jp; 4 Healthcare Systems Co., Ltd., Nagoya 466-0058, Aichi, Japan; ishikawa@hc-sys.jp (H.I.); yasutake@hc-sys.jp (T.Y.); 5 Preventive Medical Center, Shin Oyama City Hospital, Oyama 323-0827, Tochigi, Japan; m-nakano@hospital.oyama.tochigi.jp (M.N.); h-shinohara@hospital.oyama.tochigi.jp (H.S.); 6 Metabologenomics, Inc., Tsuruoka 997-0052, Yamagata, Japan; toru.kudo@metagen.co.jp (T.K.); yuichiro.nishimoto@metagen.co.jp (Y.N.); mizuguchi@metagen.co.jp (Y.M.); 7 Department of Food, Aroma and Cosmetic Chemistry, Faculty of Bioindustry, Tokyo University of Agriculture, Abashiri 099-2493, Hokkaido, Japan; a3endou@nodai.ac.jp; 8 Department of Food and Nutritional Sciences, College of Bioscience and Biotechnology, Chubu University, Kasugai 487-8501, Aichi, Japan; shimomura@isc.chubu.ac.jp

**Keywords:** insulin resistance, obesity, prebiotics, gut microbiota, *Bifidobacterium*

## Abstract

Insulin resistance leads to the onset of medical conditions such as type 2 diabetes, and its development is associated with the alteration in the gut microbiota. Although it has been demonstrated that supplementation with prebiotics modulates the gut microbiota, limited evidence is available for effects of prebiotics on insulin resistance, especially for humans. We investigated the prebiotic effect of 1-kestose supplementation on fasting insulin concentration in obesity-prone humans and rats. In the preliminary study using rats, the hyperinsulinemia induced by high-fat diet was suppressed by intake of water with 2% (*w*/*v*) 1-kestose. In the clinical study using obese-prone volunteers, the fasting serum insulin level was significantly reduced from 6.5 µU/mL (95% CI, 5.5–7.6) to 5.3 (4.6–6.0) by the 12-week intervention with supplementation of 10 g 1-kestose/day, whereas it was not changed by the intervention with placebo (6.2 µU/mL (5.4–7.1) and 6.5 (5.5–7.6) before and after intervention, respectively). The relative abundance of fecal *Bifidobacterium* was significantly increased to 0.3244 (SD, 0.1526) in 1-kestose-supplemented participants compared to that in control participants (0.1971 (0.1158)). These results suggest that prebiotic intervention using 1–kestose may potentially ameliorate insulin resistance in overweight humans via the modulation of the gut microbiota. UMIN 000028824.

## 1. Introduction

Insulin resistance, defined as an impaired stimulation of insulin to targeted tissues, is a risk factor for a wide range of disorders and clinical concerns, including hypertension, type 2 diabetes, and cardiovascular disease [1,2]. It is well-known that high-fat diets (HFDs) induce insulin resistance in association with obesity in rodents [3,4]. In recent years, insulin resistance is increasingly recognized as an association with the gut microbiota and resultant metabolites in mice and humans [5,6]. Cohort studies reported that microbially-derived metabolites are associated with the deterioration of glucose metabolism induced by obesity and type 2 diabetes [7,8,9].

In obese rodents, diet components but not obese states are the dominant determinant of the gut microbial composition [10]. Thus, dietary interventions targeting the gut microbiota composition using prebiotics and probiotics have been studied in animals and clinical trials whether the interventions ameliorate insulin resistance in the obese state (reviewed in [11,12,13,14]). Administration of probiotic *Lactobacillus* and *Bifidobacterium* lessened insulin resistance in HFD-fed mice [15]. In humans, a multi-probiotic, *Bifidobacterium*, *Lactobacillus*, *Lactococcus*, and *Propionibacterium* lowered insulin resistance as estimated by the homeostasis model assessment-estimated insulin resistance (HOMA-IR) in patients with type 2 diabetes [16]. Administration of prebiotics is also a promising approach to alleviate the development of insulin resistance in humans [17]. For example, a prebiotic, inulin, ameliorated insulinemia in obese participants with obesity-related metabolic disorders such as hypertension [18]. However, evidence for the role of prebiotics in preventing the development of insulin resistance in obese-prone adult humans is still limited.

1-Kestose is the smallest unit of fructooligosaccharides (FOS), whose structure is a fructose monomer linked with sucrose via β-2,1 glycosidic bonds [19,20]. Previous studies indicated that 1-kestose could raise insulin sensitivity in rats [21] and that 1-kestose suppressed the development of glucose intolerance in type 2 diabetes rats [22]. Such beneficial outcomes were accompanied by an increased population of host-beneficial microbes, including *Bifidobacterium* and butyrate-producing *Clostridium* cluster XIVa, and increased microbially derived metabolites of acetate and butyrate, increased in rat cecal content [21,22]. However, impacts of 1-kestose on insulin resistance have not been characterized in human intervention studies.

Here, we investigated the effect of 1-kestose supplementation on glucose metabolism, i.e., fasting insulin level and glucose intolerance, in HFD-fed rats as a preliminary study, and then conducted a clinical trial to examine the feasibility of 1-kestose to improve insulin resistance in obesity-prone participants.

## 2. Materials and methods

### 2.1. Rodent Studies

#### 2.1.1. Animals, Diets, and Experimental Design

All procedures were approved by the Animal Care Committee of the Nagoya University Graduate School of Bioagricultural Sciences (approval number, 2018031362).

Twenty-four male Sprague-Dawley rats aged 7 weeks were obtained from Japan SLC (Hamamatsu, Japan) and individually housed in wire-mesh cages under the previously described conditions [22]. After acclimatization to the animal room for 1 week, rats were randomly allocated to two groups and fed either a control diet (CD; D12450J (Research Diets, New Brunswick, NJ, USA, *n* = 12)) or a 60 kcal% high-fat diet (HFD; D12492 (Research Diets), *n* = 12). Rats in each group were then further divided into with or without 1-kestose (KES) subgroups (*n* = 6). 1-Kestose (purification > 95%, B Food Science Co., Ltd., Aichi, Japan) dissolved to 2% (*w*/*v*) in tap water was given to the KES subgroup during the experiment. In each subgroup, rats were provided free access to the corresponding experimental diets and water (with or without KES) for 19 weeks. During the experiment, body weight was measured weekly, and food and water intakes were measured twice a week.

Oral glucose tolerance test (OGTT) was conducted in the middle of the experimental period, as previously described [22].

About 1 week before the final day of the experiment, tail blood from rats fasted overnight (10 P.M.–10 A.M.) was collected into heparinized tubes to prepare post-heparin plasma for determination of glucose and insulin. On the final day of the experiment, the blood samples, ceca, and cecal contents were obtained, and the pH of the cecal contents was measured as previously described [22]. Other tissues (Supplementary Table S2) were removed and weighed.

#### 2.1.2. Analyses of Blood Components

Glucose in the plasma obtained during OGTT and from fasted rats was measured using a kit, Glucose CII test WAKO (WAKO, Osaka, Japan), and insulin in the plasma from fasted rats was determined using a Rat Insulin ELISA Kit (TMB) (Shibayagi, Gunma, Japan).

Analyses of the components (Supplementary Table S3) in plasma or serum obtained on the final day of the experiment were performed by Special Reference Laboratories (SRL) (Tokyo, Japan) using standard clinical methods [23,24].

#### 2.1.3. Statistical Analysis

Animal study data were analyzed using Prism (Prism 9.0.0) software (GraphPad, San Diego, CA, USA). Data are presented as mean ± standard error (SE). For multiple comparisons, two-way ANOVA with the Tukey-Kramer test was used. Differences were considered significant at *p* < 0.05.

### 2.2. Clinical Trial

#### 2.2.1. Study Design

This clinical trial was a randomized, double-blind, parallel-group, and placebo-controlled trial. This study was approved by the Ethics Committee of Healthcare Systems Co., Ltd., and the trial was registered at the University hospital Medical Information Network (UMIN) center under identification number UMIN 000028824.

#### 2.2.2. Participants

Healthy male and female participants were recruited in July 2017 from Oyama, Tochigi, Japan, to participate in the clinical study at Shin-Oyama City Hospital. The participants were eligible if they met the following inclusion criteria at the screening visit: (1) aged between 20 and 64 years and (2) with a body mass index (BMI) ≥ 23 kg/m^2^. Exclusion criteria were: (1) clinical systemic and metabolic disease including diabetes under treatment with medication and/or known to have a medical history, (2) HOMA-IR ≥ 2.5, (3) fasting blood glucose ≥ 126 mg/dL, (4) low-density lipoprotein (LDL) cholesterol ≥ 140 mg/dL, (5) blood triglyceride (TG) ≥ 150 mg/dL, (6) known to have food allergies, (7) participation in another biomedical trial during the past 30 days, or planned within the clinical trial, and (8) pregnancy in progress or planned within the clinical trial, or initiated breastfeeding. Food and beverage intakes of participants were assessed using 3-day food records before entering the trial.

Participants were provided written informed consent at the screening visit and randomly assigned to either the kestose or placebo group stratified by age and HOMA-IR value. HOMA-IR was calculated with fasting plasma insulin (μU/mL) × fasting plasma glucose (mg/dL)/405.

#### 2.2.3. Outcomes

The primary outcome of this study was the effect of 1-kestose on blood insulin concentration in participants classified as overweight/obese I but otherwise healthy [25]. An association of the outcome on the blood insulin concentrations with the gut microbiota composition was considered as the secondary outcome. Other secondary endpoints were anthropometric measurements and fecal and blood biomarkers.

#### 2.2.4. Study Protocol

The participants included in the 12-week intervention were randomly assigned to consume either 5 g twice per day (at breakfast and dinner) of 1-kestose (B Food Science Co., Ltd., Chita, Japan) or 5 g twice per day (at breakfast and dinner) of maltodextrin placebo (Hayashibara Co., Ltd., Okayama, Japan), provided in identical packaging. The participants were asked to finish eating by 8 P.M., not to drink anything other than water between dinner and the next morning’s meal, and not to use medication or quasi-drugs that might interfere with glucose homeostasis, any type of dietary supplements including prebiotics, and/or artificial nutritional support. Otherwise, the participants were instructed to maintain their usual lifestyles. On the first day of the trial, the participants were instructed to complete the brief-type self-administered diet history questionnaire (BDHQ) [26]. Participants were asked to record in study diaries, including daily intake of 1-kestose or maltodextrin, any sign of illness, use of medication, deviation of the study protocol, and other complaints.

#### 2.2.5. Anthropometric Characteristics

Height was measured at the screening visit. Weight, blood pressure, and pulse were measured, and BMI was calculated at baseline (Week 0), and Weeks 4, 8, and 12, and at Week 16 as a follow-up assessment.

#### 2.2.6. OGTT

Participants underwent a 75 g oral glucose tolerance test at baseline and Week 12. After sampling the baseline value of OGTT, participants were instructed to ingest a beverage containing 75 g of glucose. Blood samples were taken at 30, 60, 90, and 120 min post-ingestion. Serum glucose values were determined by an enzymatic method (LSI Medience, Tokyo, Japan). Insulin concentrations were determined by CLIA (LSI Medience). The Trapezoidal Rule was used to determine the area under the curve (AUC) for glucose and insulin.

#### 2.2.7. Serum Analysis

Venous blood samples were collected at baseline, the end of Weeks 4, 8, and 12, and the end of Week 16 as a follow-up visit. Glucose, Hemoglobin A1c (HbA1c), insulin, total cholesterol, LDL cholesterol, high-density lipoprotein (HDL) cholesterol, triglycerides, aspartate aminotransferase (AST), alanine aminotransferase (ALT), γ-glutamyl transferase (γGT), urea nitrogen, creatinine, uric acid, albumin, total protein, alkaline phosphatase (ALP), lactate dehydrogenase (LDH), sodium, chloride, potassium, calcium, inorganic phosphate, and magnesium were measured in fasting blood samples using standard clinical methods. Serum analyses were conducted by LSI Medience (Tokyo, Japan).

#### 2.2.8. Gut Microbiota Composition

Stool samples of participants were collected at baseline and the end of Week 12 with a DNA stabilizer (FS-0009, TechnoSuruga, Shizuoka, Japan) according to the manufacturer’s instructions and then stored at −80 °C until use. DNA extraction from stool samples, library construction, and 16S rRNA gene sequencing were conducted by TechnoSuruga Laboratory (Shizuoka, Japan) as described [22]. QIIME2 (version 2019.10) was used for 16S rRNA gene analysis [27]. In the analytical pipeline, sequence data were processed by using ‘cutadapt’ software (version 2.6) [28] for primer trimming (option: --p-discard-untrimmed) and ‘DADA2′ pipeline for quality filtering and denoising (options: --p-trunc-len-f 280 --p-trunc-len-r 220) [29]. The filtered output sequences were assigned to taxa using the “qiime feature-classifier classify-sklearn” command with the default parameters. Silva SSU Ref Nr 99 (version 132) was used as a reference database for taxonomic assignment [30].

#### 2.2.9. Statistical Analysis

Data are presented as mean (standard deviation (SD)) or median with 95% confidence interval (CI), as appropriate. Statistical analyses of primary outcomes were performed using Prism (Prism 9.0.0) software (GraphPad) or Microsoft Excel. Two-way repeated-measures ANOVA with Bonferroni’s multiple comparison test was used in statistical testing for OGTT. Unpaired *t*-tests were performed for comparison between treatment with 1-kestose and placebo. Comparisons between baseline and Week 12 values of each group were analyzed using relative change value and a paired *t*-test. Relative change values were calculated with (Week 12 value—baseline value)/baseline value. For subgroup analysis, treatment effects of 1-kestose were expressed as Cohen’s d, weighted mean differences, and their 95% CIs and forest plot [31]. Cohen’s d was automatically converted to Hedges’s g to compensate for small sample bias and prevent overestimation [32]. Pearson’s correlation coefficient was applied for correlations, using the relative change value. Differences were considered significant at *p* < 0.05.

For the secondary outcomes of the gut microbial composition in the clinical test, unweighted and weighted UniFrac distances were calculated for comprehensive analysis of gut microbiota using the “qiime phylogeny align-to-tree-mafft-fasttree” and “qiime diversity core-metrics-phylogenetic” commands (options: --p-sampling-depth 10000). Principal coordinate analysis (PCoA) was performed using scikit-learn (version 0.20.0) for visualizing the distances of gut microbiota. Permutational analysis of variance (PERMANOVA) analysis was performed using R software (version 3.6.3) with the “vegan” library (version 2.5–7). The relative abundance of each microbial taxon between placebo Week 12 and kestose Week 12 was compared by the Mann-Whitney *U* test. Likewise, relative abundances between kestose Week 12 and baseline, and placebo Week 12 and baseline were compared by Wilcoxon’s signed-rank test. For this comparison, we used in-house python scripts (Python 2.7.15) with the ‘scipy’ library (version 1.1.0) at a significance level of 0.05. In these statistical analyses, bacterial taxa exhibiting 0.001 or more relative abundance on average were targeted.

## 3. Results

### 3.1. Animal Study

#### 3.1.1. A High-Fat Diet Induces Obesity in Rats

The body weight of rats fed HFD tended to be greater than that of rats fed CD (Supplementary Table S1); the body weight of the HFD without KES group was significantly higher than that of the CD without KES group (*p* = 0.008). Although daily food intake in both groups of rats fed HFD was significantly lower than in corresponding groups of rats fed CD, daily energy intake showed the reverse trend (Supplementary Table S1). Consumption of 1-kestose was the same between CD and HFD with KES groups (Supplementary Table S1).

Perirenal adipose tissue weight was significantly higher in both groups of rats fed HFD than in the corresponding groups of rats fed CD (Supplementary Table S2). The cecal tissue weight of the CD with KES group was greater than that of the CD without KES group, but that in rats fed HFD was the same with/without KES (Supplementary Table S2). The cecal content weights showed the same trend as for cecal tissue weights. Other tissue weights were not affected by HFD and 1-kestose.

#### 3.1.2. Supplementation of 1-Kestose Improves Glucose Tolerance in Rats

**Figure 1:**
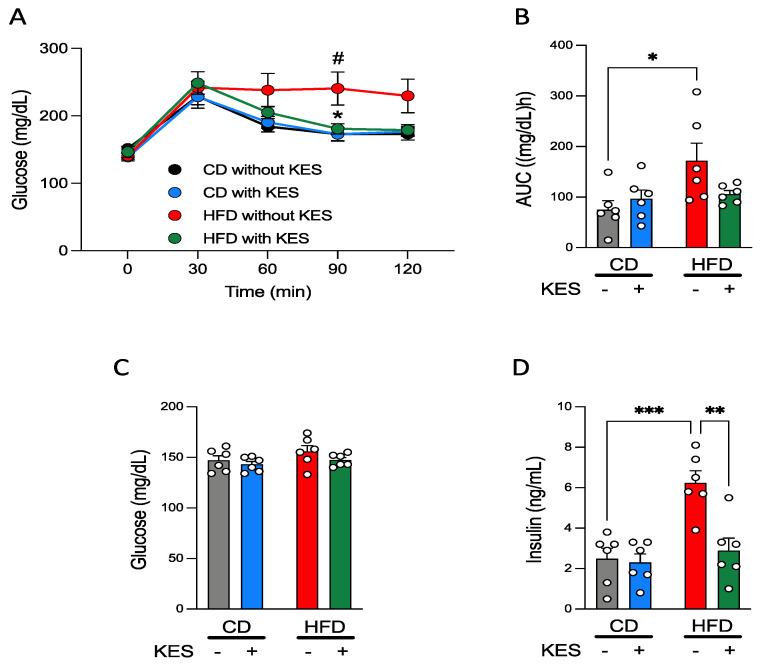
1-Kestose suppresses the development of insulin resistance. (**A**): Oral glucose tolerance tests (OGTTs) were conducted on rats fed CD or HFD with or without KES. (**B**): An AUC-glucose during OGTTs. (**C**): Fasting plasma glucose concentrations were measured after 18 weeks of the experiment. (**D**): Fasting plasma insulin concentrations were measured after 18 weeks of the experiment. *p* values were determined using two-way ANOVA with Tukey-Kramer’s test. (**A**): * *p* < 0.05 HFD with KES vs. HFD without KES. ^#^ *p* < 0.05 HFD without KES vs. CON without KES. (**B**,**D**): * *p* < 0.05, ** *p* < 0.01, and *** *p* < 0.001. Abbreviations: CD, control diet; HFD, high-fat diet; KES, 1-kestose; AUC, area under the curve.

The patterns of plasma glucose concentrations during OGTT were the same between the 2 groups of rats fed CD and showed a peak at 30 min after glucose administration. However, there were clear differences between the 2 groups of rats fed HFD; the glucose concentration during OGTT in the HFD without KES group peaked at 30 min after glucose administration, and the high level was sustained during OGTT, whereas the pattern of plasma glucose concentration in HFD with KES group was almost the same as observed for rats fed CD (Figure 1A). The plasma glucose concentration at 90 min during OGTT was significantly higher in the HFD without KES group than in the HFD with KES and CD without KES groups (Figure 1A). Although the AUC of the plasma glucose concentration during OGTT (AUC-glucose) was significantly higher in the HFD without KES group than in the CD without KES group, the AUC-glucose in the HFD with KES group was similar in level as those in rats fed CD (Figure 1B).

#### 3.1.3. Concentrations of Plasma Glucose and Insulin under Fasting and Fed States in Rats

Blood samples under the fasting state were collected about 1 week before the final day of the experiment and subjected to measurement of plasma glucose and insulin concentrations. Fasting plasma glucose concentration was not altered by both HFD and 1-kestose (Figure 1C). The concentration of fasting plasma insulin was significantly increased only in the HFD without KES group compared to the other groups (Figure 1D).

Rats were sacrificed on the final day of the experiments under fed conditions. Although plasma glucose concentrations were not significantly different among the 4 groups, the plasma insulin concentration of the HFD without KES group was relatively higher than that of CD without KES (*p* = 0.0501), and significantly higher than that of HFD with KES (Supplementary Table S3). Serum TG concentration was significantly decreased in both groups of rats fed HFD compared to the corresponding groups of rats fed CD, and other components in serum were not affected by HFD and 1-kestose (Supplementary Table S3).

### 3.2. Interventional Human Study

#### 3.2.1. Characteristics of Participants

We conducted a randomized, double-blind, parallel-group, and placebo-controlled trial in pre-diabetic participants. One hundred and eighteen participants were enrolled and assessed at the screening visit. Of the participants, 50 participants were included in the present study based on the inclusion and exclusion criteria described in the Methods. Participants were provided signed consent to participate in the study and were randomly assigned to either the kestose or placebo group stratified by age and HOMA-IR value. Of these, 4 participants discontinued the study due to personal reasons during the study. Forty-six participants completed the study; however, one participant did not sufficiently take the assigned package (<80% of total packages), and 7 participants did not follow the protocol. Therefore, we excluded the 8 participants from the analysis. A total of 38 participants, 20 in the kestose and 18 in the placebo groups, were included in the analyses (Supplementary Figure S1).

Baseline characteristics of the kestose and placebo groups are shown in Table 1; both groups were comparable, with no significant differences between the two groups of any of the variables determined in the present study. None of the participants was diagnosed with a chronic disease, including diabetes and cardiovascular diseases, at the screening. The average participant BMI of the participants was 26.1 kg/m^2^ (95% CI, 24.8–27.4) and 26.1 kg/m^2^ (95% CI, 24.9–27.2) in the kestose and placebo groups, respectively, revealing that most of the participants were categorized as either overweight or obese I according to the definition of obesity in Japan [25,33].

During the study, blood samples of participants were collected, and outcomes are shown in Supplementary Table S4. The parameter of ALT was slightly affected by supplementation of 1-kestose. Adverse events reported in the groups are described in Supplementary Table S5. Marked changes were not observed in the follow-up assessment at Week 16 (Supplementary Table S6), except for serum potassium concentration.

#### 3.2.2. Serum Insulin Concentration

**Figure 2:**
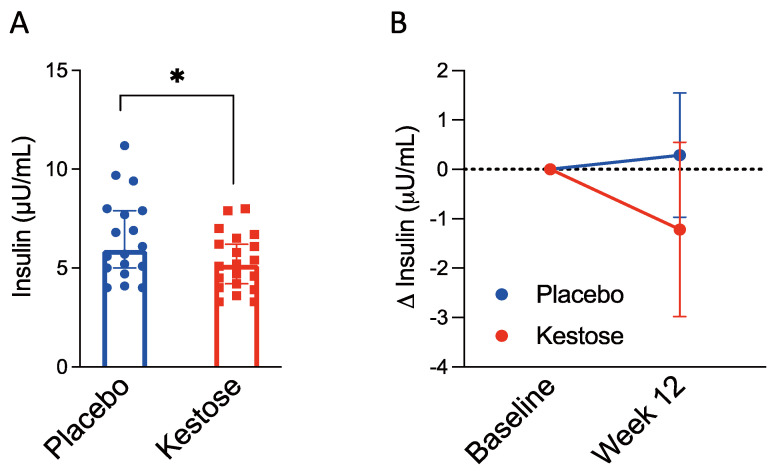
Fasting serum insulin level was lower in the kestose group compared to the placebo group. (**A**): Fasting serum insulin concentration was measured after the 12-week intervention with or without 1-kestose. (**B**): Differences of insulin concentrations during the 12-week intervention with 95% CI were calculated. Abbreviations: CI, confidence interval. (**A**,**B**) were assessed for statistical significance using Student’s *t*-test. * *p* < 0.05.

Supplementation of 1-kestose significantly reduced serum insulin concentration (mean (95% CI)) at Week 12 compared to that in the placebo group (5.3 µU/mL (4.6–6.0) vs. 6.5 µU/mL (.5–7.6), *p* < 0.05) (Figure 2A). We calculated ∆ insulin concentrations during the 12-week intervention and found a tendency to reduce in the kestose group by a mean of −1.2 (95% CI, -3.0–0.5, *p* = 0.16), although the placebo group showed almost no change (Figure 2B). Furthermore, subgroup analyses revealed that 1-kestose significantly reduced insulin concentrations in the male (5.2 µU/mL (4.3–6.2) vs. 6.9 µU/mL (5.5–8.3), *p* < 0.05) and 23–43 year groups (4.7 µU/mL (3.5–5.9) vs. 7.7 µU/mL (5.7–9.7), *p* < 0.01) (Supplementary Table S7), which was confirmed in the treatment effect using kestose/placebo comparisons of Δ insulin (Week 12—baseline) (Supplementary Figure S2). A higher negative correlation between baseline insulin value and relative value of Δ insulin (Week 12—baseline) was shown in the kestose group than in the placebo group (Pearson r, −0.76 (*p* < 0.0001) vs. −0.52 (*p* = 0.03)) (Supplementary Table S8).

In the results of OGTT at Week 12, the glucose concentration at each time point was not significantly different between the placebo and kestose groups (Table 2). However, when the glucose concentrations were compared between baseline and Week 12, the concentration at 120 min was significantly lower only in Week 12 of the kestose group (100.4 mg/dL (90.4–110.4) vs. 115.8 mg/dL (102.0–129.5), *p* < 0.05) (Table 2). The insulin concentration tended to be lower at 30 min in Week 12 OGTT in the kestose group than in the placebo group, although the difference was not statistically significant (*p* = 0.051).

#### 3.2.3. Supplementation of 1-Kestose Alters Gut Microbial Composition

Data obtained by 16S rRNA sequencing of stool samples collected at baseline and the end of the intervention (Week 12) were used to study the impact of kestose supplementation on gut microbiota composition. The PCoA plot based on the sequencing data shows that 1-kestose intervention led to microbial profile separation from the placebo (Supplementary Figure S3A). The observed results were supported by PERMANOVA analysis (Supplementary Figure S3A). No significant differences were observed in Shannon diversity between the placebo and kestose groups at Week 12 (Supplementary Figure S3B).

**Figure 3:**
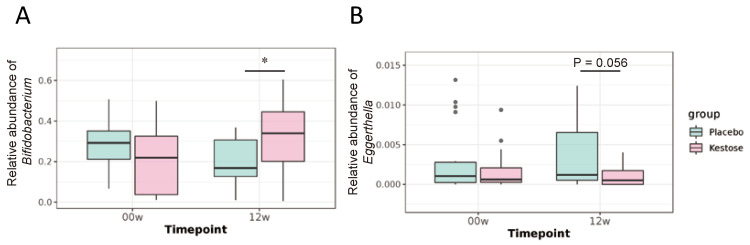
The relative abundance of *Bifidobacterium* increased in the kestose group after the 12-week intervention. (**A**): The relative abundance of genus Bifidobacterium was expressed as the median with the interquartile range at baseline and Week 12. (**B**): The relative abundance of genus *Eggerthella* was expressed as the median with the interquartile range at baseline and Week12. (**A**,**B**) were assessed for statistical significance using the Kruskal-Wallis test. * *p* < 0.05.

Relative abundance of *Bifidobacterium* was significantly increased in the kestose group at Week 12 compared to the placebo group (Figure 3A; Table 3), whereas the relative abundances of genera *Blautia*, *Sellimonas*, and *Erysipelatoclostridum* were reduced compared to those in the placebo at Week 12 (Table 3). Furthermore, 1-kestose supplementation showed a reduced tendency of the genus *Eggerthella* at Week 12 compared to that in the placebo group (0.0011 vs. 0.0037, *p* = 0.056) (Figure 3B; Table 3). Compared to the baseline, the relative abundances of several genera at Week 12, including *Streptococcus*, were reduced in the kestose group, whereas those of *Megasphaera* and *Lactobacillus* were increased in the kestose group (Table 3).

## 4. Discussion

Accumulating studies have linked the incidence of insulin resistance with the composition of the gut microbiota. Thus far, evidence for the role of prebiotics in preventing the development of insulin resistance in obesity-prone adult humans has been scarcely reported. The present study showed the potential of 1-kestose to lessen the risk of developing insulin resistance in rodents and humans with an overweight/obese state. In HFD-fed rats, we found that 1-kestose supplementation improved clinical signs of insulin resistance, i.e., glucose intolerance and hyperinsulinemia. These results agree with previous findings on the effect of dietary FOS including 1-kestose on insulin concentrations in type 2 diabetic model rats [3,22,34].

1-Kestose also reduced plasma insulin concentrations in obesity-prone adults after a 12-week intervention, although the present clinical study included the limitation as the modest sample size. The effect may have been attributed to considerable variation in baseline characteristics and microbiota between the participants. The correlation analysis revealed a negative correlation between baseline insulin concentration and Δ insulin (Week 12—baseline), suggesting that when the baseline plasma insulin concentration was relatively low, the impact of 1-kestose on glucose homeostasis was moderate. The subgroup analysis of the present human study revealed that the effect of 1-kestose was more robust in the 23–43 year subgroup than the 44+ subgroup. The insulin-lowering effect of 1-kestose in young adults is in agreement with other studies using prebiotic inulin and oligosaccharides [35,36], suggesting that age affects the effect of 1-kestose on plasma insulin concentration, and the younger generation (23–43 years old) was more susceptible to 1-kestose. Differences in gut microbiota according to age and sex, especially with respect to the population of bifidobacteria [37,38], might be one of the reasons for the obtained results.

Our present study supports the previous study using a FOS mixture, i.e., 1-kestose (degree of polymerization (DP) 3), nystose (DP4), and fructosylnystose (DP5). Daily intake of 21 g of the FOS mixture positively affected plasma insulin level in overweight/obese adults, where absolute insulin concentration was significantly decreased in the FOS supplemented group between baseline and 12-week measurements [39]. In comparison, a daily intake of 10 g (5 g twice a day) of 1-kestose was sufficient to reduce the insulin concentration in the present study. The study evaluating six separate prebiotics on morbidly obese human fecal cultures reported that 1-kestose was the most active prebiotic in terms of fermentability on the gut microbiota [40]. DP4 nystose is usually the major component of commercialized FOS [13,20], whereas DP3 1-kestose showed better growth promoting activity in bifidobacteria and butyrate-producing human commensal *Anaerostipes caccae* than nystose [41]. The different activity was due to absence of genes involved in nystose-hydrolyzing enzymes and/or poor gene induction activity of nystose in the organisms [42]. Thus, FOS, especially in 1-kestose, is a promising prebiotic to alleviate insulin resistance-related disorders in obese-prone adults.

The explanation of which 1-kestose elicits insulin reduction appears to be partly due to a shift in the gut microbiota observed in the present study, such as altering the relative abundance of select genera and the global microbial community. As for the select genera, we observed that 1-kestose increased the relative abundance of *Bifidobacterium* and decreased those of *Blautia* and *Sellimonas* compared to the placebo group. The population of *Blautia* is inconsistently reported to be associated with obesity [43,44]. A meta-analysis, which re-analyzed published data of 16S amplicon sequencing of gut microbiota, revealed that depletion of health-associated bacteria, including *Bifidobacterium*, is characterized as a shared response to disease-associated microbial shift [45].

The observed reduction in the relative abundance of *Streptococcus* and the relatively low level of *Eggerthella* in the present human study may also be relevant to the insulin-lowering effect of 1-kestose supplementation. The two genera are reported to catabolize histidine to imidazole propionate, which inhibits insulin signaling, resulting in impaired glucose tolerance in mice [7,9]. The microbially derived metabolite is associated with insulin resistance in humans [8]. A reduced population of *Eggerthella* by supplementation of 1-kestose was reported in an in vitro fecal culture study using stools obtained from healthy adults and morbidly obese subjects [40,46]. Accordingly, we speculate that 1-kestose would protect hosts from the metabolite produced by the microbiota. Further studies are needed to verify this hypothesis.

Our previous and present studies found significant impacts of 1-kestose on insulin levels in both diet-induced obese rats and type 2 diabetes rats. 1-Kestose reduced plasma insulin concentration by the 12-week intervention independent of other lifestyle changes in the human study. Of note, participants with relatively high baseline insulin levels showed higher efficacy of 1-kestose, suggesting that dietary 1-kestose might be further used for clinical application in patients with type 2 diabetes. Our results support a role for 1-kestose in impacting the gut environment by modulating select genera and host health. A better understanding of prebiotic effects should enable the design of improved dietary supplements for preventing the development of insulin resistance.

## Figures and Tables

**Table 1:** Baseline characteristics.

Characteristics	Placebo		Kestose		*p* Value
	(*n* = 18)		(*n* = 20)		
Sex (male/female)	10/8		14/6		
Age (years)	43.4	(11.7)	45.2	(9.5)	0.76
Weight (kg)	72.6	(9.4)	75.5	(9.5)	0.33
Hight (cm)	166.8	(8.5)	170.1	(7.2)	0.21
BMI (kg/m^2^) *	26.1	(2.3)	26.1	(2.8)	0.86
HbA1c (%)	5.4	(0.3)	5.4	(0.3)	0.77
HbA1c (mmol/mol)	35.1	(2.9)	35.0	(3.3)	
Glucose (mg/dL)	85.9	(5.5)	87.2	(11.4)	0.59
Insulin (µU/mL)	6.2	(1.7)	6.5	(4.0)	0.49
HOMA-IR ^†^	1.3	(0.4)	1.5	(1.2)	0.35
Total chol (mg/dL)	191.3	(18.1)	201.1	(27.4)	0.16
LDL chol (mg/dL)	110.7	(15.5)	120.9	(21.4)	0.11
HDL chol (mg/dL)	55.6	(10.2)	55.5	(12.8)	0.97
TG (mg/dL)	89.6	(40.5)	88.4	(31.3)	0.91
AST (U/L)	18.8	(5.4)	20.2	(7.3)	0.97
ALT (U/L)	18.3	(9.5)	26.7	(22.9)	0.22
γGT (U/L)	39.9	(44.8)	38.8	(21.5)	0.36
ALP (U/L)	210.3	(57.9)	212.2	(57.7)	0.98
BUN (mg/dL)	12.9	(2.9)	13.8	(2.7)	0.40
Creatinine (mg/dL)	0.7	(0.1)	0.8	(0.1)	0.22
Uric acid (mg/dL)	5.0	(1.5)	5.7	(1.2)	0.20
Albumin (g/dL)	4.4	(0.3)	4.4	(0.3)	0.75
Total protein (g/dL)	7.5	(0.4)	7.4	(0.4)	0.47
LDH (U/L)	180.0	(28.8)	175.6	(29.8)	0.60
Sodium (mEq/L)	142.7	(2.1)	143.0	(1.9)	0.82
Chloride (mEq/L)	102.0	(2.0)	102.2	(2.4)	0.84
Potassium (mEq/L)	4.2	(0.3)	4.0	(0.4)	0.48
Calcium (mg/dL)	9.4	(0.4)	9.4	(0.3)	0.89
I. Ph (mg/dL)	4.1	(1.1)	4.0	(0.9)	0.87
Mg (mg/dL)	2.2	(0.1)	2.2	(0.2)	0.92

Data are presented as mean (SD). Abbreviations: BMI, body mass index; HbA1c, hemoglobin A1c; HOMA-IR, Homeostatic Model Assessment of Insulin Resistance; chol, cholesterol; LDL, low-density lipoprotein; HDL, high-density lipoprotein; TG, triglycerides; AST, aspartate aminotransferase; ALT, alanine aminotransferase; γGT, gamma-glutamyltransferase; ALP, alkaline phosphatase; LDH, lactate dehydrogenase; BUN, blood urea nitrogen; I. Ph; inorganic phosphate; Mg, magnesium. *p* values are calculated using Student’s *t*-test. * BMI is calculated as weight in kilograms divided by the square of height in meters. ^†^ HOMA-IR is calculated as fasting insulin in µU/L multiplied by fasting glucose in nmol/L and then divided by 405.

**Table 2:** Oral glucose tolerance tests at baseline and Week 12.

Clinical Test Item	Placebo (*n* = 18)				Kestose (*n* = 20)			
	Baseline		Week 12		Baseline		Week 12	
**Glucose (mg/dL)**								
0 min	85.9	(5.5)	86.7	(7.5)	87.2	(11.4)	88.6	(8.3)
30 min	136.0	(21.3)	132.1	(19.7)	145.1	(23.4)	142.4	(28.6)
60 min	137.7	(35.8)	132.4	(46.7)	140.5	(46.7)	151.3	(45.1)
90 min	122.3	(28.3)	118.5	(32.9)	126.6	(38.5)	121.4	(35.9)
120 min	109.4	(19.4)	105.5	(20.6)	115.8	(29.3)	100.4	(21.3) *
AUC [(mg/dL)h]	75.1	(37.2)	67.7	(43.0)	84.3	(48.0)	78.1	(48.2)
**Insulin (µU/mL)**								
0 min	6.2	(1.7)	6.5	(2.1)	6.5	(4.0)	5.3	(1.4)
30 min	46.5	(35.2)	45.9	(23.8)	43.9	(33.7)	29.2	(13.2)
60 min	45.5	(21.0)	49.2	(24.2)	39.7	(21.7)	42.7	(19.9)
90 min	40.3	(17.6)	38.1	(19.9)	35.6	(21.3)	35.9	(21.0)
120 min	35.5	(18.1)	36.6	(13.5)	34.8	(22.0)	36.5	(27.9)
AUC [(µU/mL)min]	128.3	(58.9)	128.7	(53.3)	113.9	(55.7)	107.5	(52.0)

Data are presented as mean (SD). Abbreviation: AUC, area under the curve. * *p* < 0.05 vs. Baseline value of the kestose group.

**Table 3:** Relative abundance of gut microbiota at baseline and Week 12.

	Placebo (*n* = 18)							Kestose (*n* = 20)							
	Baseline			Week 12				Baseline			Week 12				
	**Relative abundance**														
*Blautia*	0.1665	±	0.0777	0.1984	±	0.0800		0.1530	±	0.0777	0.1281	±	0.0656		^††^
*Bifidobacterium*	0.2723	±	0.1201	0.1971	±	0.1158	^*^	0.2209	±	0.1762	0.3244	±	0.1526	^*^	^††^
*Sellimonas*	0.0031	±	0.0072	0.0044	±	0.0097		0.0009	±	0.0022	0.0006	±	0.0012		^†^
*Erysipelatoclostridium*	0.0022	±	0.0030	0.0028	±	0.0034		0.0007	±	0.0010	0.0008	±	0.0014		^†^
*Megasphaera*	0.0019	±	0.0051	0.0025	±	0.0081		0.0098	±	0.0281	0.0143	±	0.0364	^*^	
*Streptococcus*	0.0414	±	0.0478	0.0451	±	0.0654		0.0379	±	0.0429	0.0183	±	0.0179	^*^	
*Ruminiclostridium 5*	0.0028	±	0.0044	0.0085	±	0.0137	^**^	0.0091	±	0.0204	0.0054	±	0.0119		
*Ruminococcaceae* UCG-013	0.0052	±	0.0088	0.0081	±	0.0125	^*^	0.0039	±	0.0046	0.0054	±	0.0081		
*Lachnospiraceae* NK4A136 group	0.0006	±	0.0014	0.0029	±	0.0044	^**^	0.0040	±	0.0078	0.0014	±	0.0023		
*Lactobacillus*	0.0034	±	0.0065	0.0040	±	0.0088		0.0020	±	0.0076	0.0142	±	0.0533	^**^	
*Turicibacter*	0.0035	±	0.0083	0.0045	±	0.0122		0.0034	±	0.0064	0.0017	±	0.0047	^*^	
*Bacillus*	0.0004	±	0.0009	0.0095	±	0.0153	^*^	0.0051	±	0.0092	0.0038	±	0.0050		
[*Ruminococcus*] *gnavus* group	0.0065	±	0.0132	0.0142	±	0.0349		0.0149	±	0.0238	0.0064	±	0.0084	^**^	
*Collinsella*	0.0953	±	0.0530	0.0796	±	0.0485	^*^	0.0676	±	0.0414	0.0792	±	0.0571		
*Christensenellaceae* R-7 group	0.0020	±	0.0046	0.0045	±	0.0144		0.0050	±	0.0130	0.0033	±	0.0099	^*^	
*Eggerthella*	0.0031	±	0.0043	0.0037	±	0.0043		0.0017	±	0.0024	0.0011	±	0.0015		

Data are presented as mean ± SD. * *p* < 0.05 and ** *p* < 0.01 vs. Baseline value of the intragroup. ^†^ *p* < 0.05 and ^††^ *p* < 0.01 vs. placebo value at 12 weeks.

## Data Availability

The data from this study are available upon request from the corresponding author.

## Supplementary Materials

The following are available online: https://www.mdpi.com/article/10.3390/nu13092983/s1. Figure S1: Flow chart of subject eligibility.; Figure S2: A forest plot represents treatment effects on relative changes of insulin concentrations during the 12-week intervention with or without 1-kestose.; Figure S3: An altered gut microbial composition between placebo and ketose groups with the 12-week intervention.; Table S1: Body weight and food intake of rats fed HFD with or without KES.; Table S2: Tissue weights of rats fed HFD with or without KES.; Table S3: Concentrations of blood components in rats fed HFD with or without KES.; Table S4: Characteristics of participants during the intervention with or without 1-kestose.; Table S5: Adverse events throughout the study.; Table S6: Characteristics of the participants at Week 16.; Table S7: Insulin concentration (µU/mL) of subgroups.; Table S8: Correlation analysis between inulin baseline value and relative changes of insulin values during the 12-week intervention with or without 1-kestose.
