# Supplementary material for: Supplementation of 1-Kestose Modulates the Gut Microbiota Composition to Ameliorate Glucose Metabolism in Obesity-Prone Hosts
Source: Nutrients. 2021 Aug 27;13(9):2983. doi: 10.3390/nu13092983 (PMC8470827; doi:10.3390/nu13092983)
Supplement: Supplementary file 1 [file nutrients-13-02983-s001.zip › supplementary-9.8.docx]

**Supplementary Table S1.** Body weight and food intake of rats fed HFD with or without KES

|  | CD | | | | | | HFD | | | | | | |  |
| --- | --- | --- | --- | --- | --- | --- | --- | --- | --- | --- | --- | --- | --- | --- |
| KES | - | | | + | | | - | | |  | + | | |  |
| Body weight (g) | 677 | ± | 16 | 690 | ± | 26 | 806 | ± | 36 | ^#^ | 731 | ± | 23 |  |
| Food intake (g/day) | 23.7 | ± | 0.3 | 23.7 | ± | 0.8 | 20.5 | ± | 0.9 | ^#^ | 18.7 | ± | 0.5 | ^$^ |
| Energy intake (kcal/day) | 94.9 | ± | 1.3 | 94.8 | ± | 3.2 | 107.6 | ± | 4.2 | ^#^ | 98.0 | ± | 2.7 | ^$^ |
| Water intake (g/day) | 22.4 | ± | 1.7 | 21.0 | ± | 0.6 | 24.5 | ± | 2.0 |  | 21.6 | ± | 1.3 |  |
| 1-Kestose intake  (g/day) | 0.0 | ± | 0.0 | 0.4 | ± | 0.0 | 0.0 | ± | 0.0 |  | 0.4 | ± | 0.0 |  |

Data are presented as mean ± SE. n = 6 animals per group.

Abbreviations: CD, control diet; HFD, high-fat diet; KES, 1-kestose.

^#^*P* < 0.05 vs. CD without KES, and ^$^*P* < 0.05 vs. CD with KES. *P* values are calculated using the Tukey-Kramer’s test.

**Supplementary Table S2.** Tissue weights of rats fed HFD with or without KES

|  | CD | | | | | |  | HFD | | | | | | |  |
| --- | --- | --- | --- | --- | --- | --- | --- | --- | --- | --- | --- | --- | --- | --- | --- |
| KES | - | | | + | | |  | - | | |  | + | | |  |
| Heart (g) | 1.4 | ± | 0.1 | 1.4 | ± | 0.0 |  | 1.4 | ± | 0.0 |  | 1.3 | ± | 0.0 |  |
| Liver (g) | 25.5 | ± | 1.5 | 24.3 | ± | 1.4 |  | 29.3 | ± | 2.5 |  | 22.9 | ± | 1.0 |  |
| Kidney (g) | 3.4 | ± | 0.1 | 3.3 | ± | 0.2 |  | 3.8 | ± | 0.3 |  | 3.3 | ± | 0.1 |  |
| Perirenal adipose tissue (g) | 22.8 | ± | 1.0 | 24.4 | ± | 2.6 |  | 37.2 | ± | 2.4 | ^#^ | 37.4 | ± | 1.6 | ^$^ |
| Epididymal adipose tissue (g) | 20.8 | ± | 0.5 | 20.6 | ± | 1.9 |  | 26.9 | ± | 2.1 |  | 30.3 | ± | 1.9 | ^$^ |
| Cecal tissue (g) | 0.9 | ± | 0.0 | 1.1 | ± | 0.1 | ^†^ | 0.9 | ± | 0.1 |  | 0.9 | ± | 0.1 | ^$^ |
| Cecal content (g) | 2.6 | ± | 0.3 | 3.1 | ± | 0.3 |  | 2.2 | ± | 0.1 |  | 2.4 | ± | 0.2 |  |

Data are presented as mean ± SE. n = 6 animals per group.

Abbreviations: CD, control diet; HFD, high-fat diet; KES, 1-kestose.

^#^*P* < 0.05 vs. CD without KES, and ^$^*P* < 0.05 vs. CD with KES. *P* values are calculated using the Tukey-Kramer’s test.

**Supplementary Table** S**3.** Concentrations of blood components in rats fed HFD with or without KES

|  | CD | | | | | | HFD | | | | | | |  |
| --- | --- | --- | --- | --- | --- | --- | --- | --- | --- | --- | --- | --- | --- | --- |
| KES | - | | | + | | | - | | |  | + | | |  |
| Plasma |  |  |  |  |  |  |  |  |  |  |  |  |  |  |
| Glucose (mg/dL) | 200.0 | ± | 6.6 | 211.3 | ± | 13.9 | 211.5 | ± | 8.3 |  | 186.8 | ± | 8.0 |  |
| Insulin (ng/mL) | 3.4 | ± | 0.6 | 3.3 | ± | 0.4 | 5.6 | ± | 0.8 |  | 3.1 | ± | 0.2 | ^*^ |
| Serum |  |  |  |  |  |  |  |  |  |  |  |  |  |  |
| TG (mg/dL) | 266.0 | ± | 33.0 | 209.0 | ± | 16.0 | 171.0 | ± | 16.0 | ^#^ | 118.0 | ± | 16.0 | ^$^ |
| FFAs (μmol/L) | 822.0 | ± | 75.0 | 795.0 | ± | 57.0 | 1082.3 | ± | 77.4 |  | 1057.0 | ± | 160.0 |  |
| Total chol (mg/dL) | 140.0 | ± | 20.0 | 137.0 | ± | 11.0 | 125.0 | ± | 12.0 |  | 114.0 | ± | 9.0 |  |
| HDL chol (mg/dL) | 26.0 | ± | 3.0 | 25.0 | ± | 1.0 | 26.0 | ± | 1.0 |  | 26.0 | ± | 1.0 |  |
| LDL chol (mg/dL) | 15.0 | ± | 2.0 | 15.0 | ± | 1.0 | 16.0 | ± | 2.0 |  | 13.0 | ± | 1.0 |  |

Data are presented as mean ± SE. n = 6 animals per group.

Abbreviations: CD, control diet; HFD, high-fat diet; KES, 1-kestose; TG, triglycerides; FFAs, free fatty acids; HDL, high-density lipoprotein; chol, cholesterol; LDL, low-density lipoprotein.

^#^*P* < 0.05 vs. CD without KES, ^$^*P* < 0.05 vs. CD with KES, and *P < 0.05 vs. HFD without KES. *P* values are calculated using the Tukey-Kramer’s test.

**Supplementary Table S4.** Characteristics of participants during the intervention with or without 1-kestose

|  | Placebo (n = 18) | | | | | | | | Kestose (n = 20) | | | | | | | | Two-way ANOVA | | |
| --- | --- | --- | --- | --- | --- | --- | --- | --- | --- | --- | --- | --- | --- | --- | --- | --- | --- | --- | --- |
|  | Baseline | | Week 4 | | Week 8 | | Week 12 | | Baseline | | Week 4 | | Week 8 | | Week 12 | | KES | Time | Interaction |
| Weight (kg) | 72.6 | (9.4) | 72.7 | (10.0) | 72.6 | (9.9) | 73.1 | (9.8) | 75.5 | (9.5) | 75.7 | (9.7) | 75.9 | (9.3) | 76.0 | (9.4) | ns | ns | ns |
| BMI (kg/m2) | 26.1 | (2.3) | 26.1 | (2.5) | 26.1 | (2.4) | 26.3 | (2.5) | 26.1 | (2.8) | 26.2 | (2.9) | 26.2 | (2.7) | 26.3 | (2.7) | ns | ns | ns |
| HbA1c (%) | 5.36 | (0.26) | 5.43 | (0.24) | 5.34 | (0.21) | 5.33 | (0.23) | 5.35 | (0.30) | 5.45 | (0.29)^***^ | 5.32 | (0.28) | 5.32 | (0.27) | ns | <0.001 | ns |
| HbA1c (mmol/mol) | 35.1 | (2.9) | 35.9 | (2.7) | 34.8 | (2.3) | 34.7 | (2.6) | 35.0 | (3.3) | 36.0 | (3.2) | 34.6 | (3.0) | 34.6 | (2.9) |  |  |  |
| Glucose (mg/dL) | 85.9 | (5.5) | 83.4 | (6.2) | 88.2 | (6.7) | 86.7 | (7.5) | 87.2 | (11.4) | 86.0 | (6.8) | 90.1 | (6.5) | 88.6 | (8.3) | ns | <0.05 | ns |
| Insulin (µU/mL) | 6.2 | (1.7) | 5.7 | (1.7) | 7.1 | (2.6) | 6.5 | (2.1) | 6.5 | (4.0) | 5.5 | (2.0) | 6.1 | (2.3) | 5.3 | (1.4) | ns | ns | ns |
| HOMA-IR | 1.3 | (0.4) | 1.2 | (0.4) | 1.6 | (0.6) | 1.4 | (0.5) | 1.5 | (1.2) | 1.2 | (0.5) | 1.4 | (0.5) | 1.2 | (0.4) | ns | ns | ns |
| Total chol (mg/dL) | 191.3 | (18.1) | 201.6 | (24.6) | 198.2 | (21.5) | 196.4 | (22.4) | 201.1 | (27.4) | 213.0 | (24.6)^*^ | 207.8 | (27.0) | 206.4 | (24.0) | ns | <0.01 | ns |
| LDL chol (mg/dL) | 110.7 | (15.5) | 119.6 | (19.1)^*^ | 118.8 | (19.1)^*^ | 112.8 | (16.8) | 120.9 | (21.4) | 128.0 | (19.5) | 125.7 | (19.0) | 123.2 | (16.7) | ns | <0.01 | ns |
| HDL chol (mg/dL) | 55.6 | (10.2) | 59.3 | (13.6) | 58.2 | (10.7) | 58.0 | (11.8) | 55.5 | (12.8) | 60.6 | (15.3)^**^ | 57.7 | (15.1) | 60.3 | (14.9)^**^ | ns | <0.01 | ns |
| TG (mg/dL) | 89.6 | (40.5) | 90.9 | (33.2) | 90.5 | (43.6) | 96.3 | (50.1) | 88.4 | (31.3) | 95.8 | (42.1) | 95.9 | (35.3) | 85.1 | (30.8) | ns | ns | ns |
| AST (U/L) | 18.8 | (5.4) | 20.4 | (4.6) | 19.7 | (5.4) | 19.6 | (4.5) | 20.2 | (7.3) | 24.6 | (12.3) | 21.1 | (6.4) | 22.8 | (6.7) | ns | <0.05 | ns |
| ALT (U/L) | 18.3 | (9.5) | 19.7 | (8.9) | 18.0 | (7.1) | 18.9 | (7.4) | 26.7 | (22.9) | 31.3 | (19.3) | 26.9 | (15.2) | 27.3 | (15.7) | <0.05 | ns | ns |
| γGT (U/L) | 39.9 | (44.8) | 42.8 | (44.5) | 42.5 | (46.9) | 43.4 | (48.6) | 38.8 | (21.5) | 47.8 | (33.9) | 45.1 | (31.5) | 44.4 | (35.2) | ns | ns | ns |
| ALP (U/L) | 210.3 | (57.9) | 214.8 | (54.2) | 222.9 | (61.5) | 215.5 | (56.5) | 212.2 | (57.7) | 222.7 | (60.0) | 226.0 | (66.3) | 227.3 | (70.6) | ns | <0.05 | ns |
| LDH (U/L) | 180.0 | (28.8) | 178.8 | (23.2) | 171.8 | (23.9) | 175.6 | (21.9) | 175.6 | (29.8) | 178.4 | (33.1) | 171.5 | (25.1) | 180.8 | (30.4) | ns | <0.05 | ns |
| BUN (mg/dL) | 12.9 | (2.9) | 12.4 | (2.8) | 12.6 | (3.0) | 13.1 | (2.7) | 13.8 | (2.7) | 12.9 | (2.8) | 13.6 | (2.9) | 13.8 | (2.6) | ns | ns | ns |
| Creatinine (mg/dL) | 0.7 | (0.1) | 0.7 | (0.1) | 0.8 | (0.2) | 0.7 | (0.2) | 0.8 | (0.1) | 0.8 | (0.1) | 0.8 | (0.1) | 0.8 | (0.1) | ns | ns | ns |
| Uric acid (mg/dL) | 5.0 | (1.5) | 5.1 | (1.3) | 5.3 | (1.3) | 5.0 | (1.3) | 5.7 | (1.2) | 5.8 | (0.9) | 5.8 | (1.0) | 5.7 | (1.0) | ns | ns | ns |
| Albumin (g/dL) | 4.4 | (0.3) | 4.5 | (0.2) | 4.5 | (0.3) | 4.4 | (0.3) | 4.4 | (0.3) | 4.5 | (0.3) | 4.5 | (0.4) | 4.5 | (0.3) | ns | ns | ns |
| Total protein (g/dL) | 7.5 | (0.4) | 7.6 | (0.5) | 7.5 | (0.4) | 7.4 | (0.4) | 7.4 | (0.4) | 7.5 | (0.3) | 7.3 | (0.4) | 7.4 | (0.4) | ns | ns | ns |
| Sodium (mEq/L) | 142.7 | (2.1) | 142.1 | (1.9) | 142.3 | (1.6) | 141.8 | (2.4) | 143.0 | (1.9) | 142.7 | (1.9) | 142.4 | (1.7) | 142.8 | (2.0) | ns | ns | ns |
| Chloride (mEq/L) | 102.0 | (2.0) | 102.6 | (1.9) | 103.9 | (1.7)^**^ | 102.6 | (1.5) | 102.2 | (2.4) | 102.2 | (1.9) | 103.4 | (2.3) | 102.5 | (2.9) | ns | <0.001 | ns |
| Potassium (mEq/L) | 4.2 | (0.3) | 4.7 | (0.4)^***^ | 4.6 | (0.4)^***^ | 4.4 | (0.4) | 4.0 | (0.4) | 4.6 | (0.5)^***^ | 4.3 | (0.4)^†,*^ | 4.2 | (0.4) | ns | <0.001 | ns |
| Calcium (mg/dL) | 9.4 | (0.4) | 9.5 | (0.2) | 9.4 | (0.3) | 9.3 | (0.3) | 9.4 | (0.3) | 9.5 | (0.3) | 9.2 | (0.4)^*^ | 9.4 | (0.4) | ns | <0.01 | ns |
| I.Ph (mg/dL) | 4.1 | (1.1) | 3.0 | (0.5)^**^ | 3.2 | (0.5)^*^ | 3.2 | (0.7)^**^ | 4.0 | (0.9) | 2.7 | (0.4)^***^ | 3.1 | (0.4)^**^ | 3.0 | (0.5)^***^ | ns | <0.001 | ns |
| Mg (mg/dL) | 2.2 | (0.1) | 2.2 | (0.1) | 2.2 | (0.1) | 2.2 | (0.2) | 2.2 | (0.2) | 2.2 | (0.1) | 2.2 | (0.1) | 2.2 | (0.1) | ns | ns | ns |

Data are presented as mean (SD).

Abbreviations: BMI, body mass index; HbA1c, hemoglobin A1c; HOMA-IR, Homeostatic Model Assessment of Insulin Resistance; chol, cholesterol; LDL, low-density lipoprotein; HDL, high-density lipoprotein; TG, triglyceride; AST, aspartate aminotransferase; ALT, alanine aminotransferase; γGT, gamma-glutamyl transferase; ALP, alkaline phosphatase; LDH, lactate dehydrogenase; BUN, blood urea nitrogen; I. Ph; inorganic phosphate; Mg, magnesium.

* *P* < 0.05, ** *P* < 0.01, and *** *P* < 0.001 vs. baseline values of the intragroup. ^†^ *P* < 0.05 vs. the same time point value of the intergroup. *P* values are calculated using Bonferroni’s multiple comparison test.

^†^BMI is calculated as weight in kilograms divided by the square of height in meters.

^‡^HOMA-IR is calculated as fasting insulin in µU/L multiplied by fasting glucose in nmol/L and then divided by 405.

**Supplementary Table S5.** Adverse events throughout the study.

|  | Placebo | Kestose |
| --- | --- | --- |
| Abdominal pain | 6 | 0 |
| Headache | 1 | 4 |
| Influenza | 1 | 1 |
| Colon cancer | 0 | 1 |
| Lymphoedema | 1 | 0 |
| Chills | 0 | 1 |

A total of 16 adverse events throughout the study were reported, and three adverse events were reported after the 12-week intervention. One adverse event, colon cancer, was reported as moderate. All other adverse events were mild in intensity. Four events, including headache and influenza, required medical intervention, and three events, including headache and chills, were needed treatment with nonprescription medicine. None of the adverse events except for colon cancer were of long-term duration. There were no discontinuations because of adverse events.

**Supplementary Table S6.** Characteristics of the participants at Week 16.

|  | Placebo (n = 18) | | Kestose (n = 20) | | *P* value |
| --- | --- | --- | --- | --- | --- |
| Sex (male/female) | 10/8 | | 14/6 | |  |
| Weight (kg) | 73.4 | (10.0) | 76.1 | (9.3) | 0.30 |
| BMI (kg/m^2^)^†^ | 26.4 | (2.4) | 26.3 | (2.7) | 0.93 |
| HbA1c (%) | 5.4 | (0.2) | 5.4 | (0.3) | 0.68 |
| HbA1c (mmol/mol) | 35.3 | (2.6) | 35.1 | (2.8) |  |
| Glucose (mg/dL) | 87.1 | (8.6) | 89.4 | (9.2) | 0.40 |
| Insulin (µU/mL) | 6.5 | (2.1) | 6.8 | (2.6) | 0.82 |
| HOMA-IR^‡^ | 1.4 | (0.5) | 1.5 | (0.6) | 0.76 |
| Total chol (mg/dL) | 203.4 | (19.1) | 215.9 | (24.4) | 0.09 |
| LDL chol (mg/dL) | 125.3 | (19.8) | 133.6 | (19.6) | 0.32 |
| HDL chol (mg/dL) | 58.6 | (13.1) | 62.6 | (14.2) | 0.31 |
| TG (mg/dL) | 112.9 | (115.8) | 95.9 | (56.7) | 0.79 |
| AST (U/L) | 20.8 | (4.2) | 23.0 | (7.4) | 0.80 |
| ALT (U/L) | 21.7 | (8.4) | 30.8 | (17.8) | 0.05 |
| γGT (U/L) | 41.2 | (40.9) | 50.8 | (35.7) | 0.29 |
| ALP (U/L) | 201.6 | (50.4) | 215.2 | (57.0) | 0.81 |
| BUN (mg/dL) | 13.3 | (3.0) | 13.3 | (3.4) | 0.93 |
| Creatinine (mg/dL) | 0.7 | (0.2) | 0.8 | (0.1) | 0.27 |
| Uric acid (mg/dL) | 5.1 | (1.6) | 5.8 | (1.1) | 0.20 |
| Albumin (g/dL) | 4.3 | (0.3) | 4.5 | (0.3) | 0.07 |
| Total protein (g/dL) | 7.4 | (0.3) | 7.3 | (0.3) | 0.45 |
| LDH (U/L) | 177.2 | (29.4) | 173.7 | (27.7) | 0.70 |
| Sodium (mEq/L) | 140.1 | (2.5) | 139.3 | (2.3) | 0.33 |
| Chloride (mEq/L) | 103.6 | (1.5) | 103.0 | (2.6) | 0.58 |
| Potassium (mEq/L) | 6.5 | (0.7) | 5.8 | (0.6) | <0.001 |
| Calcium (mg/dL) | 9.1 | (0.3) | 9.1 | (0.4) | 0.82 |
| I. Ph (mg/dL) | 3.1 | (0.4) | 3.0 | (0.3) | 0.75 |
| Mg (mg/dL) | 2.2 | (0.1) | 2.2 | (0.1) | 0.99 |

The potassium value was slightly reduced in the kestose group compared to the placebo group. Data are presented as mean (SD).

Abbreviations: BMI, body mass index; HbA1c, hemoglobin A1c; HOMA-IR, Homeostatic Model Assessment of Insulin Resistance; chol, cholesterol; LDL, low-density lipoprotein; HDL, high-density lipoprotein; TG, triglycerides; AST, aspartate aminotransferase; ALT, alanine aminotransferase; γGT, gamma-glutamyl transferase; ALP, alkaline phosphatase; LDH, lactate dehydrogenase; BUN, blood urea nitrogen; I. Ph; inorganic phosphate; Mg, magnesium.

*P* values were calculated using Student’s t-test.

^†^BMI is calculated as weight in kilograms divided by the square of height in meters.

^‡^HOMA-IR is calculated as fasting insulin in µU/L multiplied by fasting glucose in nmol/L and then divided by 405.

**Supplementary Table S7.** Insulin concentration (µU/mL) of subgroups.

|  | Placebo (n = 18) | | | | | | |  | Kestose (n = 20) | | | | | | | |
| --- | --- | --- | --- | --- | --- | --- | --- | --- | --- | --- | --- | --- | --- | --- | --- | --- |
|  | Baseline | | Week 12 | | Changes | |  |  | Baseline | | Week 12 | |  | Changes | |  |
|  | mean (SD) | | | | | | n (%) |  | mean (SD) | | | | | | | n (%) |
| Sex |  |  |  |  |  |  |  |  |  |  |  |  |  |  |  |  |
| Male | 5.7 | (1.5) | 6.9 | (1.9) | 1.2 | (2.3) | 10 (55.6) |  | 6.3 | (4.6) | 5.2 | (1.6) | ^☨^ | -1.0 | (4.5) | 14 (70) |
| Female | 6.9 | (1.8) | 6.1 | (2.4) | -0.9 | (2.5) | 8 (44.4) |  | 7.1 | (1.8) | 5.5 | (1.1) |  | -1.7 | (1.2) | 6 (30) |
| Ages |  |  |  |  |  |  |  |  |  |  |  |  |  |  |  |  |
| 23-43 years | 6.7 | (2.0) | 7.7 | (2.4) | 1.0 | (3.5) | 8 (44.4) |  | 7.1 | (5.9) | 4.7 | (1.4) | ^☨☨^ | -2.4 | (5.6) | 8 (40) |
| 44+ years | 5.8 | (1.4) | 5.5 | (1.2) | -0.3 | (1.4) | 10 (55.6) |  | 6.1 | (2.1) | 5.7 | (1.4) |  | -0.4 | (1.6) | 12 (60) |

Abbreviation: SD, standard deviation.

^☨^*P* < 0.05 and ^☨☨^*P* < 0.01 vs. the placebo value of the same time point. *P* values are calculated using Student’s t-test.

**Supplementary Table S8.** Correlation analysis between inulin baseline value and relative changes of insulin values during the 12-week intervention with or without 1-kestose.

|  | Relative change in insulin value^*^ | | | |
| --- | --- | --- | --- | --- |
|  | Placebo (n = 18) | | Kestose (n = 20) | |
|  | PCC | *P* value | PCC | *P* value |
| Insulin baseline value | -0.52 | 0.03 | -0.76 | <0.0001 |

^*^Relative change of insulin value is calculated as (Week 12 value - baseline value)/baseline value.

Abbreviation: PCC, Pearson’s correlation coefficient.

**Supplementary Figure S1** Flow chart of subject eligibility.

**Supplementary Figure S2** A forest plot represents treatment effects on relative changes of insulin concentrations during the 12-week intervention with or without 1-kestose. Each subgroup size is represented by a solid circle, square, triangle, and rhombus. Treatment effects were calculated by Cohen’s d, which was converted to Hedges’s g to compensate for small sample bias and prevent overestimation. Abbreviation: CI, confidence interval.

**Supplementary Figure S3** An altered gut microbial composition between placebo and ketose groups with the 12-week intervention. *A*: PCoA plot and PERMANOVA results of β-diversity from fecal microbes of placebo and kestose groups at baseline and Week 12. *B*: Shannon diversity index in fecal 16S rRNA amplicon sequencing data at baseline and Week 12. *B* was assessed for statistical significance using the Kruskal-Wallis test.
