# Supplementary figures and images for: Supplementation of 1-Kestose Modulates the Gut Microbiota Composition to Ameliorate Glucose Metabolism in Obesity-Prone Hosts
Source: Nutrients. 2021 Aug 27;13(9):2983. doi: 10.3390/nu13092983 (PMC8470827; doi:10.3390/nu13092983)

Supplementary Figure S1

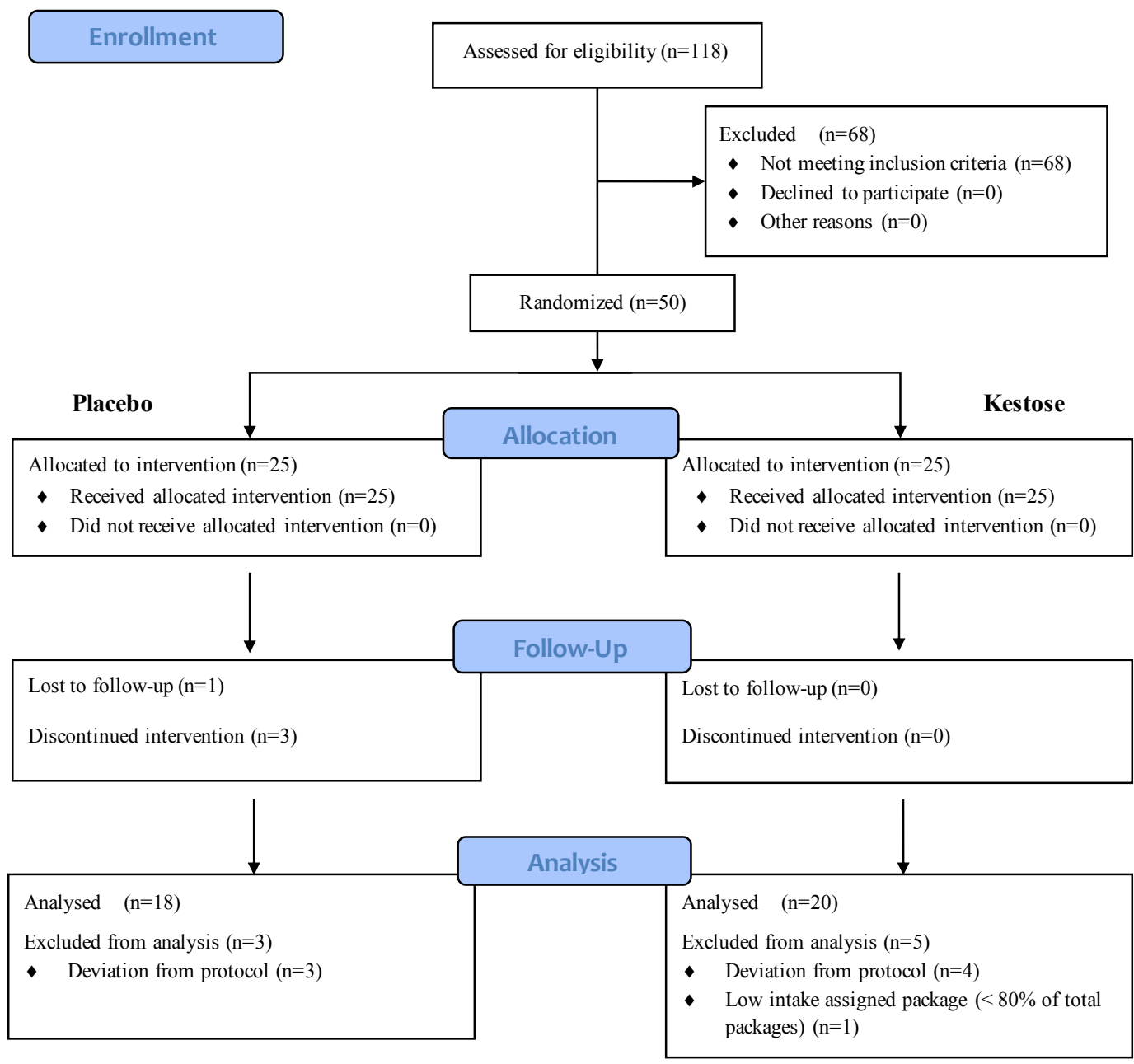

Supplementary Figure S2

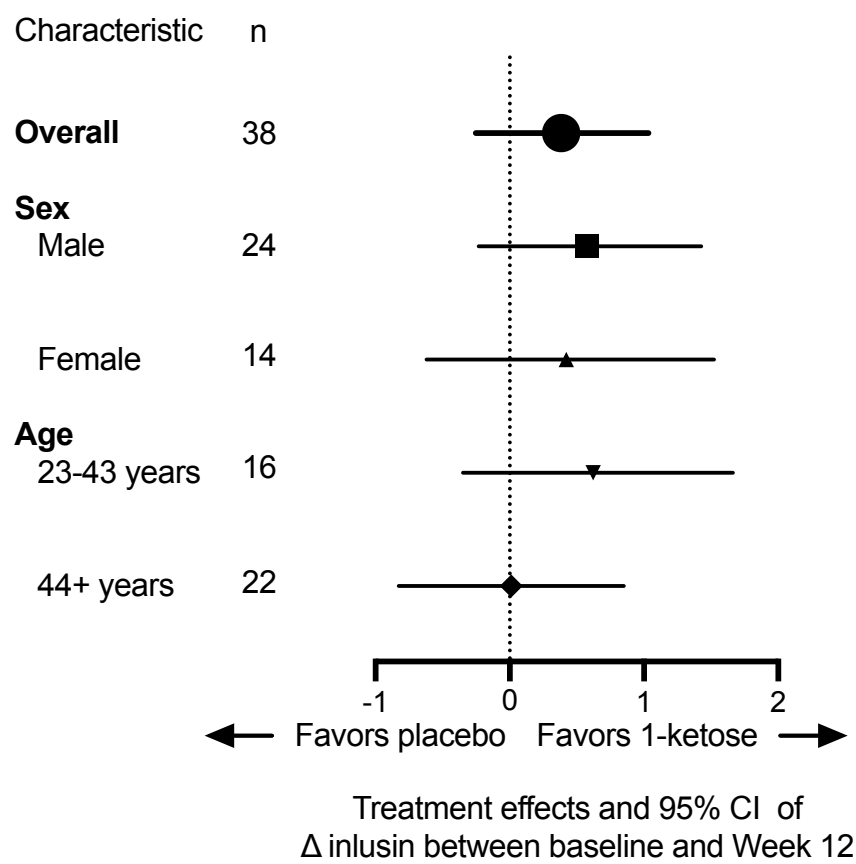

# Supplementary Figure S3

A

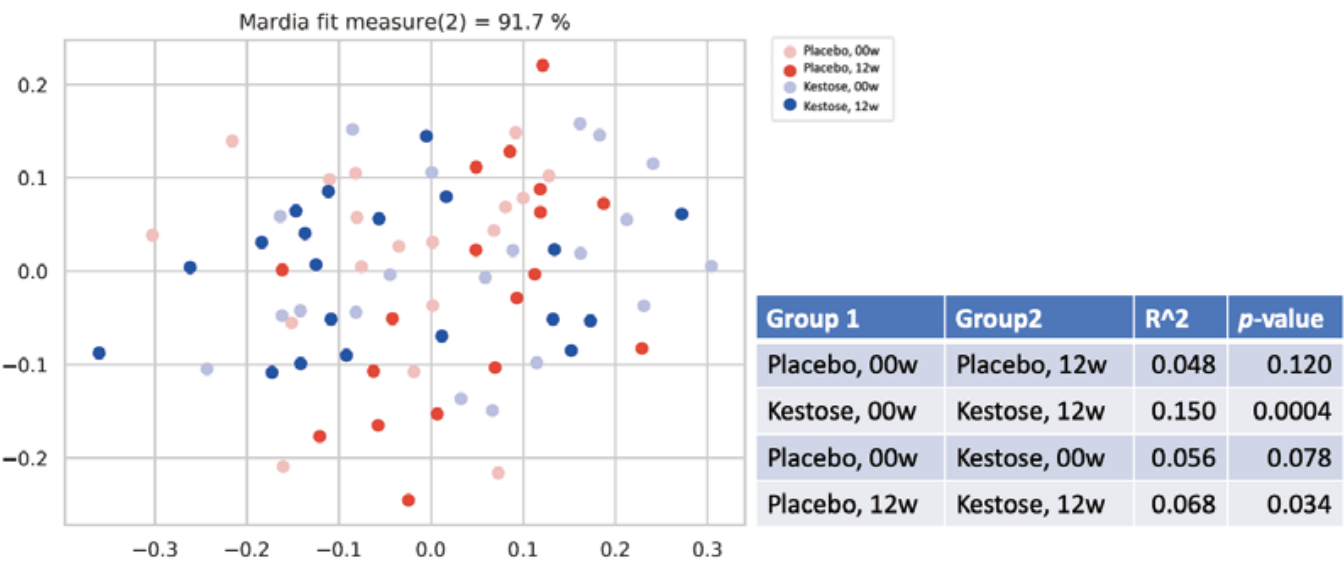

B

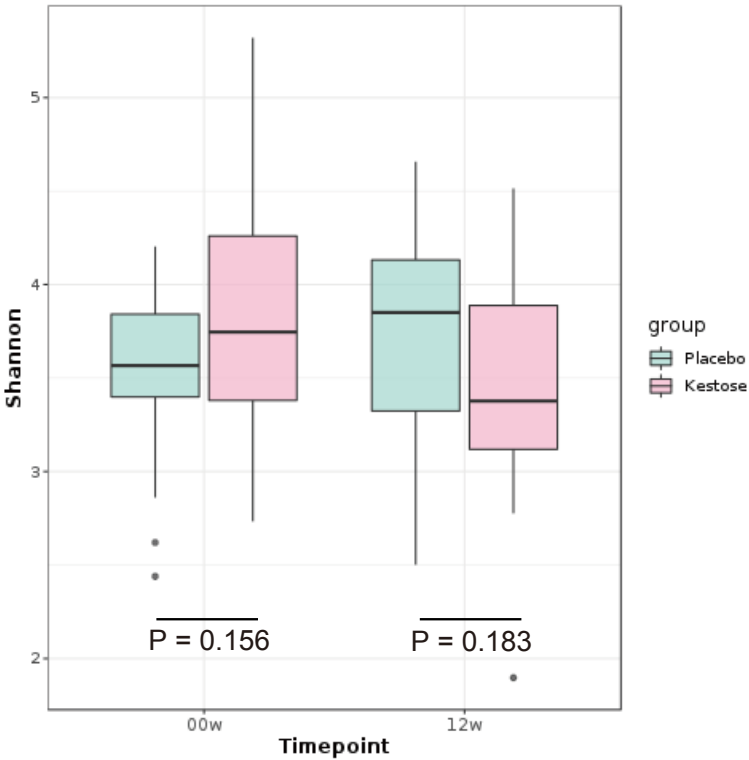

Supplement: Supplementary file 1 [file nutrients-13-02983-s001.zip › Supplementary-9.8.pdf]
